# Molecular mechanisms of two-component system RhpRS regulating type III secretion system in *Pseudomonas syringae*

**DOI:** 10.1093/nar/gku865

**Published:** 2014-09-23

**Authors:** Xin Deng, Haihua Liang, Kai Chen, Chuan He, Lefu Lan, Xiaoyan Tang

**Affiliations:** 1 Department of Chemistry and Institute for Biophysical Dynamics, University of Chicago, Chicago, IL 60637, USA; 2 Department of Life Science, Northwest University, Xi'an, Shaanxi 710069, China; 3 Shanghai Institute of Materia Medica, Chinese Academy of Sciences, 555 Zuchongzhi Road, Pudong Zhangjiang Hi-Tech Park, Shanghai 201203, China; 4 College of Life Sciences, Capital Normal University, Beijing 100048, China

## Abstract

*Pseudomonas syringae* uses the two-component system RhpRS to regulate the expression of type III secretion system (T3SS) genes and bacterial virulence. However, the molecular mechanisms and the regulons of RhpRS have yet to be fully elucidated. Here, we show that RhpS functions as a kinase and a phosphatase on RhpR and as an autokinase upon itself. RhpR is phosphorylated by the small phosphodonor acetyl phosphate. A specific RhpR-binding site containing the inverted repeat (IR) motif GTATC-N_6_-GATAC, was mapped to its own promoter by a DNase I footprint analysis. Electrophoretic mobility shift assay indicated that P-RhpR has a higher binding affinity to the IR motif than RhpR. To identify additional RhpR targets in *P. syringae*, we performed chromatin immunoprecipitation followed by high-throughput DNA sequencing (ChIP-seq) and detected 167 enriched loci including the *hrpR* promoter, suggesting the direct regulation of T3SS cascade genes by RhpR. A genome-wide microarray analysis showed that, in addition to the T3SS cascade genes, RhpR differentially regulates a large set of genes with various functions in response to different growth conditions. Together, these results suggested that RhpRS is a global regulator that allows *P. syringae* to sense and respond to environmental changes by coordinating T3SS expression and many other biological processes.

## INTRODUCTION

Many Gram-negative pathogenic bacteria rely on the needle-like type III secretion system (T3SS) to secrete a cocktail of effectors that facilitate bacterial infection of eukaryotic host organisms (1). In the plant pathogen *Pseudomonas syringae*, the T3SS genes are clustered in a chromosomal *hrp* island, which is responsible for pathogenicity on susceptible plants and the hypersensitive response on resistant and nonhost plants (2). The *hrp* island harbors genes encoding the structural components of the T3SS and regulatory proteins controlling the expression of the T3SS genes (3).

The expression of T3SS genes is coordinately regulated by many endogenous regulatory proteins in response to various environmental factors (4) (Supplementary Figure S1). The *hrp* genes are expressed at a very low levels in nutrient rich media such as King's broth (KB), but are activated rapidly in *hrp*-inducing minimal medium (MM) and in the plants (5). In *P. syringae*, the *hrp* genes are activated by an alternate sigma factor HrpL that recognizes an *hrp* box motif in the *hrp* gene promoters (6,7). In turn, the induction of *hrpL* depends on the sigma factor RpoN (σ^54^) and two NtrC-family transcription factors, HrpR and HrpS (8–10). RpoN controls the transcription of *hrpL* under a σ^54^-dependent promoter (8). The *hrpR* and *hrpS* genes are in the same operon (10,11). *hrpS* alone induces a very low level of *hrpL* expression, but the full activation of *hrpL* requires both genes (9,12). HrpR and HrpS carry an enhancer-binding motif and a module that associates with the σ^54^-RNA polymerase. HrpR and HrpS are thought to form a heterodimer that binds to the *hrpL* promoter and induces the *hrpL* transcription via an interaction with the RpoN-RNA polymerase in T3SS-inducing conditions (9). In *P. syringae*, HrpS activity is repressed by HrpV, a T3SS negative regulator that physically interacts with HrpS (13,14). HrpV-mediated repression can be cleared by HrpG, a chaperone-like protein that interacts with HrpV and liberates HrpS from HrpV-mediated repression without changing the transcription of *hrpV* (14). The *P. syringae* HrpR protein is degraded by the adenosine triphosphate (ATP)-dependent protease Lon, which also degrades unstable or misfolded proteins involved in a variety of biological processes in bacteria (15). HrpR is unstable in KB but is stable in a *lon* mutant, leading to an elevated expression of the T3SS genes in the nutrient-rich medium (15,16). Additionally, *hrpL* is also regulated by CorR, a response regulator that also controls the expression of the phytotoxin coronatine in *P. syringae* pv. *tomato* (17). Compared with the wild-type strain, the *corR* mutant has reduced expression of the *hrpL* gene. A putative CorR-binding site is located upstream of *hrpL*, and a gel shift assay confirms the binding of CorR to this DNA motif (17).

In *P. syringae*, *hrpRS* expression is regulated by *hrpA*, *aefR* and at least two two-component systems (TCS), *gacAS* and *rhpRS* (5,17–21). A mutation in *hrpA* encoding the major T3SS pilus component severely compromises the transcription of *hrpRS* and *hrpL*, which can be restored by the overexpression of *hrpRS*. However, the mechanism by which HrpA controls *hrpRS* expression is unknown (18). AefR, a known regulator of quorum sensing and epiphytic traits, also plays a role in controlling the expression of T3SS genes, and *aefR* mutation reduces the *hrpR* promoter activity (21). The GacAS system regulates multiple biological processes in various species of bacteria, such as motility, virulence, quorum-sensing and production of toxin, antibiotics, exopolysaccharides and biofilms (22). In *P. syringae*, a mutation in the response regulator gene *gacA* severely reduces the expression of *hrpRS*, *rpoN* and *hrpL*, suggesting that *gacA* is an important T3SS regulator that is located at the top of the regulatory cascade (19). Research with *Erwinia chrysanthemi* 3937 has also demonstrated that GacA is required for the expression of the T3SS genes (20). The signal perceived by GacS and the connection between GacA and *hrpRS* remain to be elucidated.

Previously, we showed that an *rhpS* mutant of *P. syringae* displays diminished induction of the T3SS genes in MM and the host plants (5). *rhpS* is located immediately downstream of its putative cognate regulator gene *rhpR* and the two genes are organized in an operon. While the *rhpS* mutant has severely reduced expression of T3SS genes, Δ*rhpRS* (the *rhpS* and *rhpR* double deletion mutant) shows the same expression level of T3SS genes as the wild-type strain. Furthermore, overexpression of *rhpR* in Δ*rhpRS* mutant suppresses the induction of T3SS genes (5). These results indicate that RhpR is a negative regulator of T3SS genes. We also observed that RhpR induces its own promoter, and the inverted repeat (IR) element GTATC-N_6_-GATAC is required for the RhpR-dependent induction (23).

Here we show that RhpS functions as a kinase and a phosphatase on RhpR and as an autokinase upon itself. We characterized the RhpR binding site by a DNase I footprint assay. In addition, a chromatin immunoprecipitation (ChIP)-seq analysis was conducted for the *rhpS* mutant, which identified 167 RhpR-binding loci including the *hrpR* promoter, suggesting the direct regulation of the *hrpRS* operon by RhpR. Finally, we conducted whole genome microarray analyses to determine genes regulated by *rhpR* in KB and MM, indicating that *rhpRS* is a global regulator that coordinates the T3SS and many other cellular activities.

## MATERIALS AND METHODS

### Bacterial strains, culture media, plasmids and primers

The bacterial strains used in this study were the wild-type, the *rhpS* mutant and the Δ*rhpRS* mutant from *P. syringae* pv. *tomato* DC3000 strain and the *rhpS* mutant from the *P. syringae* pv. *phaseolicola* strain (5). These strains were grown in KB medium (24) containing the appropriate antibiotics to an optical density at 600 nm (OD_600_) of 2.0–2.5. The bacteria were centrifuged, washed twice with MM (50- mM KH_2_PO_4_, 7.6-mM (NH_4_)_2_SO_4_, 1.7-mM MgCl_2_, 1.7-mM NaCl and 10-mM fructose, pH 5.7) (25), resuspended in MM to an OD_600_ of 0.1–0.2 and cultured for 6 h before further experimentation. Antibiotics (in mg/l) used for the selection of *P. syringae* strains were: rifampicin, 25; kanamycin, 10; spectinomycin, 50. The plasmids and primers used are listed in Supplementary Table S1. RhpR and RhpS used in the present study are from *P. syringae* pv. *tomato*, while RhpR_psph_ is from *P. syringae* pv. *phaseolicola*.

### Measurement of *rhpR-luc*(luciferase) activities in MM

Bacteria were grown in liquid KB medium containing rifampicin and spectinomycin to an OD_600_ between 2.0 to 2.5. To induce the reporter gene *rhpR-luc* in MM, bacteria were washed twice with MM, resuspended in MM to an OD_600_ of 0.1, and incubated for 6 h to allow for the induction of *rhpR-luc* (23). The cell suspension (100 μl) was mixed with 10 μl of 0.1-mM luciferin, and the LUC activity was measured using a cooled charged-coupled device (CCD) (Roper Scientific). After LUC measurement, the bacteria were diluted and plated on KB plates in order to count CFUs. The relative LUC activity was normalized to the number of bacteria in the MM.

### Purification of recombinant RhpR, RhpS^C^, PSPTO_1669 (AckA) and RhpR_psph_ proteins

For expression of the RhpR protein, the C-terminal RhpS **(**RhpS^C^) lacking its putative N-terminal transmembrane domain, the putative acetate kinase PSPTO_1669, and the RhpR_psph_ protein, we used the ligation-independent cloning method (26). The coding regions of these genes were polymerase chain reaction (PCR)-amplified from genomic DNA with primers RhpR-EXF/R, RhpSC-EXF/R, PSPTO1669-EXF/R and RhpR_psph_-EXF/R, respectively, (Supplementary Table S1). The PCR products were treated with T4 DNA polymerase in the presence of dCTP for 30 min at room temperature. Target vector pMCSG19 (26) was digested with *Ssp*I, gel purified and then treated with T4 DNA polymerase in the presence of dGTP for 15 min at 16°C. The T4 DNA polymerase-treated plasmid vector and PCR product were gel purified, mixed, incubated for 5 min at room temperature and then transformed into *Escherichia coli* strain DH5. The resulting plasmid was transformed again into BL21 star (DE3) (Science Reagents, Inc.) containing the plasmid pRK1037 (27) that cuts maltose binding protein from the fusion protein, and the transformants were selected on LB agar plates with 100-μg/ml ampicillin and 50-μg/ml kanamycin. The BL21 star (DE3) strain carrying the plasmid was grown in LB to OD_600_ = 0.6, and then 1-mM isopropyl-β-d-thiogalactopyranoside was added. After overnight induction at 16°C, the cells were harvested and frozen at −80°C. The expressed protein was purified from the frozen cells with a HisTrap column (GE Healthcare, Inc.) by following the manufacturer's recommendations. The purified proteins were supplemented with 20% glycerol and stored at −80°C.

### EMSA assay

DNA probes were PCR-amplified using primers listed in Supplementary Table S1, then radiolabeled with T4 polynucleotide kinase (NEB) and [γ-^32^P]ATP (Perkin-Elmer). The radioactive probe (2 ng) was mixed with various amounts of the RhpR or RhpR_psph_ protein in 20 μl of gel shift buffer (10-mM Tris–HCl, pH 7.4, 50-mM KCl, 5-mM MgCl_2_, 10% glycerol, 3-μg/ml sheared salmon sperm DNA). The PSPTO_1489 promoter DNA was used for the negative control. After incubation at room temperature for 20 min, the samples were analyzed by 8% polyacrylamide gel electrophoresis (100 V for prerun, 85 V for 45 min for sample separation). The gels were dried and subjected to autoradiography on a phosphor screen (BAS-IP; Fuji). The assay was repeated at least three times with similar results.

### Phosphorylation of RhpR for EMSA

The purified RhpR protein (20 μM) was mixed with purified RhpS^C^ (1 μM) in phosphorylation buffer (10-mM Tris–HCl, pH 7.4, 50-mM KCl, 5-mM MgCl_2_, 10% glycerol). One-millimolar ATP was then added, and the mixture was incubated at room temperature for 30 min before the addition of [γ-^32^P]-labeled DNA probe. For electrophoretic mobility shift assay (EMSA) or footprint assay, the entire reaction mixture was used.

### Dye primer based DNase I footprint assay

The DNase I footprint procedures were modified from published procedures (28,29). A 404-bp fragment that encompasses bases −396 to +8 of the *rhpR* promoter region was generated by PCR with the primers rhpR-FP-6FAM and rhpR-FPR (Supplementary Table S1). Fifty nanogram of 6-FAM-labled *rhpR* promoter was incubated with varying amounts of RhpR protein ranging from 0 to 1 μM in a binding buffer (10-mM Tris–HCl, pH 7.4, 50-mM KCl, 5-mM MgCl_2_, 10% glycerol, 3-μg/ml sheared salmon sperm DNA). After several optimization experiments, the DNase I digestion was found to work best with 0.05 Kunitz units of DNase I (New England Biolabs) in a 20-μl reaction for 5 min at room temperature. The reaction was stopped by 0.25 M ethylenediaminetetraacetic acid (EDTA) and extracted with phenol-chloroform-isoamyl alcohol (25:24:1). The DNA fragments were purified with the QIAquick PCR Purification kit (Qiagen) and eluted in 15 μl distilled water. Five microliter of digested DNA was added to 4.9-μl HiDi formamide (Applied Biosystems) and 0.1-μl GeneScan-500 LIZ size standards (Applied Biosystems). The samples were analyzed with the 3730 DNA Analyzer, with G5 dye set, running an altered default genotyping module that increased the injection time to 30 s and the injection voltage to 3 kV, in the sequencing facility at the University of Chicago. Results were analyzed with Peak Scanner (Applied Biosystems).

### Phosphotransfer between P-RhpS^C^ and RhpR

RhpS^C^ (1 μM) was phosphorylated by preincubation with 10-μM [γ-^32^P]ATP in 100 μl of phosphorylation buffer for 1 h at room temperature. As a reference sample, 10 μl of the reaction was mixed with 10 μl of 2x sodium dodecyl sulphate (SDS) loading buffer and kept at room temperature. To initiate the phosphotransfer reaction, RhpR (10 μM) was added to the phosphorylated RhpS^C^. At various time points (1, 30, 60 and 120 min), 10 μl of the sample was removed and mixed with 10 μl of 2x SDS loading buffer and subject to 13% sodium dodecylsulphate-polyacrylamide gel electrophoresis (SDS-PAGE) before autoradiography.

### Production of [γ-^32^P]-acetyl phosphate that phosphorylates RhpR

[γ-^32^P]-acetyl phosphate was synthesized as described (30,31). Briefly, the reaction mixture including 10-μM putative acetate kinase PSPTO_1669 (AckA), 10 μCi of [γ-^32^P]-ATP (6000 Ci/mmol, PerkinElmer) in an acetate kinase buffer (25-mM Tris–HCl [pH 7.4], 60-mM KOAc, 10-mM MgCl_2_) was incubated at room temperature for 30 min before removing acetate kinase by using a 30 kDa cut-off column (Amicon ultra with 30 kDa cut-off, Millipore). [γ-^32^P]-acetyl phosphate was mixed with 100 μM-RhpR for 30 min at room temperature. Aliquots (20 μl) were removed from the reaction and treated as described above.

### Phosphatase assay

After [γ-^32^P]-acetyl phosphate was used to *in vitro* phosphorylate RhpR, [γ-^32^P]-RhpR (100 μM) was incubated at room temperature with 1-mM RhpS^C^ in a phosphorylation buffer. Aliquots (20 μl) were removed from the reaction and treated as described above.

### Microarray analyses

The transcriptome assays were conducted by following the procedures published previously (32). Briefly, a high-density oligonucleotide array was designed to cover 5608 predicted open reading frames (ORFs) of *P. syringae* pv. *tomato* DC3000 strain. Seventeen distinct high-quality probe pairs were designed for each ORF. First-strand cDNA was synthesized by mixing 6 μl of 200 U/μl SuperScript II (Invitrogen, Carlsbad, CA, USA) with 10 μg of total RNA template in a 60-μl reaction mixture for 55 min at 42°C and then at 72°C for 15 min. RNA was then degraded by adding 1 μl of RNase H (Invitrogen) and 1 μl RNase A (Promega). After a 10-min incubation at 37°C, the mixture was extracted with phenol, the cDNA was precipitated by ethanol. The cDNA was partially digested to 50- to 200-bp fragments by DNaseI treatment (Promega) before the 3′ end was labeled with biotin-6-ddATP and deoxynucleotidyl terminal transferase (Promega). Array hybridization, washing, and staining were performed according to the standard protocol provided by NimbleGen Systems Inc. (Madison, WI, USA). Arrays were stained with Cy3-streptavidin and then anti-streptavidin antibody before scanning with an Axon Genepix 4000B scanner at 532 nm and a resolution of 5 μm. For each probe on the array, the median signal intensity was calculated using the NimbleGen extraction software. The data were normalized by using quantile normalization (33), and gene calls were generated by using the RMA algorithm (robust multichip average) (34). The relative fluorescent intensities of each ORF were scaled by assuming a constant mean signal intensity of 1000 signal units for each array, and the intensity values of replicate measurements were averaged. For each pair-wise sample comparison, *P*-values were calculated for each ORF using the two-sided *t*-test, and the ratio was log-transformed (base 2).

### ChIP-seq

ChIP was performed as previously described (35) with minor changes. The *P. syringae* pv. *phaseolicola rhpS* mutant containing an empty pHM2 or pHM2-RhpR_psph_-HA plasmid was cultured in KB medium, and then transferred to MM supplemented with spectinomycin at the mid-log phase (OD_600_ = 0.6) and cultured for 6 h. Bacteria were treated with 1% formaldehyde for 10 min at 37°C. Cross-linking was stopped by adding 125 mM glycine. Bacterial pellets were washed twice with a Tris buffer (20-mM Tris–HCl pH 7.5, 150-mM NaCl), and then re-suspended in 500 μl IP buffer (50-mM Hepes-KOH pH 7.5, 150-mM NaCl, 1-mM EDTA, 1% Triton X-100, 0.1% sodium deoxycholate, 0.1% SDS, mini-protease inhibitor cocktail (Roche)) and sonicated the DNA to sizes of 100–300 bp. Insoluble cellular debris was removed by centrifugation and the supernatant used as the input sample in IP experiments. Both control and IP samples were washed by protein A beads (General Electric), and then incubated with 50 μl agarose-conjugated anti-HA antibodies (Sigma) in the IP buffer. Washing, crosslink reversal and purification of the ChIP DNA were conducted by following previously published protocols (35). DNA fragments (150 to 250 bp) were selected for library construction and sequencing libraries prepared using the NEXTflex™ ChIP-Seq Kit (Bioo Scientific). The libraries were sequenced using the HiSeq 2000 system (Illumina). ChIP-seq reads were mapped to the *P. syringae* pv. *phaseolicola* genome using TopHat (version 2.0.0) with two mismatches allowed (36). Only the uniquely mapped reads were kept for the subsequent analyses. The enriched peaks were identified using MACS software (version 2.0.0) (37), which was followed by Multiple EM for Motif Elicitation (MEME) analyses to generate an RhpR-binding motif (38). More than 80% of peaks are shared in two experiments. The reported peaks are found in both experiments.

### Statistical analysis

Microarray and ChIP-seq analyses were repeated twice. All other experiments were repeated at least three times. Two-tailed Student's *t*-tests were performed using Microsoft Office Excel 2011.

## RESULTS

### P-RhpR has higher binding affinity than does RhpR to the inverted repeat in its own promoter

**Figure 1.**
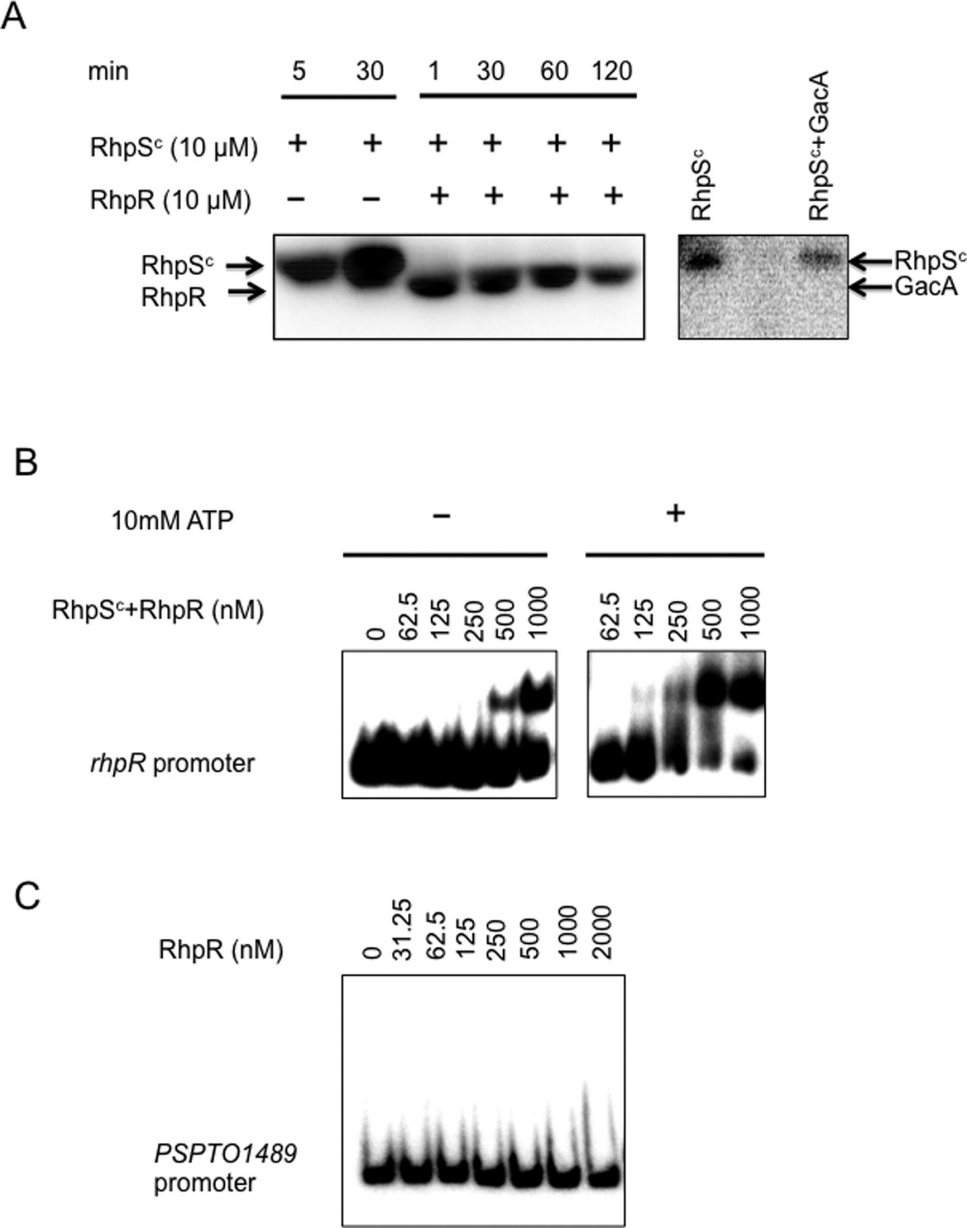
Kinase activity of RhpS^C^ and DNA binding activity of RhpR. (**A**) RhpS^C^ (2 μM) was mixed with [γ-^32^P]-ATP for 30 min before adding RhpR (10 μM) into the reaction mixture for 120 min. Phosphorylated RhpS^C^ was not able to phosphorylate non-cognate GacA over a 30 min period. (**B**) DNA binding activities of unphosphorylated RhpR (left panel) and phosphorylated RhpR (P-RhpR, right panel) to its own promoter are shown. RhpR was pre-mixed with RhpS^C^ in the presence or absence of ATP. Aliquots containing the indicated amount of RhpR protein were mixed with 2 ng of a γ-^32^P-end-labeled *rhpR* promoter fragment in EMSA buffer at room temperature for 30 min before performing a gel shift assay. (**C**) RhpR does not bind to the promoter region of PSPTO_1489.

To examine the autophosphorylation activity of RhpS and the phosphor-transfer from RhpS to RhpR, we expressed the full-length RhpS and RhpR proteins in *E. coli* with a His_6_ tag at their N-termini and then purified the proteins with an Ni-NTA column. We successfully purified the full-length RhpR, but the yield of the full-length RhpS was very low. Since the N-terminal transmembrane domain may affect the solubility of RhpS, we then expressed and purified the cytoplasmic autokinase domain of RhpS (RhpS^C^, aa 86-344) with an N-terminal His_6_ tag. The protein demonstrated strong autokinase activity and strong kinase activity toward RhpR, but not toward the TCS response regulator GacA protein (Figure 1A).

Although we previously showed that RhpR regulates multiple promoters containing an IR motif (GTATC-N_6_-GATAC, where N is any nucleotide), the direct interactions and the binding sites were not demonstrated (23). To this end, we performed EMSA assay using the purified His_6_-RhpR protein and radiolabeled *rhpR* promoter DNA. RhpR clearly bound to its own promoter in a concentration-dependent manner (Figure 1B), while there was no interaction between the control probe (the PSPTO_1489 promoter DNA) and RhpR at 2 μM (Figure 1C).

To determine whether the phosphorylation state of RhpR affects its binding activity, we performed EMSA by adding RhpS^C^ (RhpR:RhpS^C^ ratio, 10:1) and 10-mM ATP to the EMSA reaction. Phosphorylation of RhpR by RhpS^C^ requires the presence of ATP. As expected, the addition of RhpS^C^ and ATP significantly increased the binding affinity (∼8-fold, based on the free probe intensity) of RhpR to its own promoter (Figure 1B), demonstrating the importance of phosphorylation in the DNA binding affinity of RhpR.

**Figure 2.**
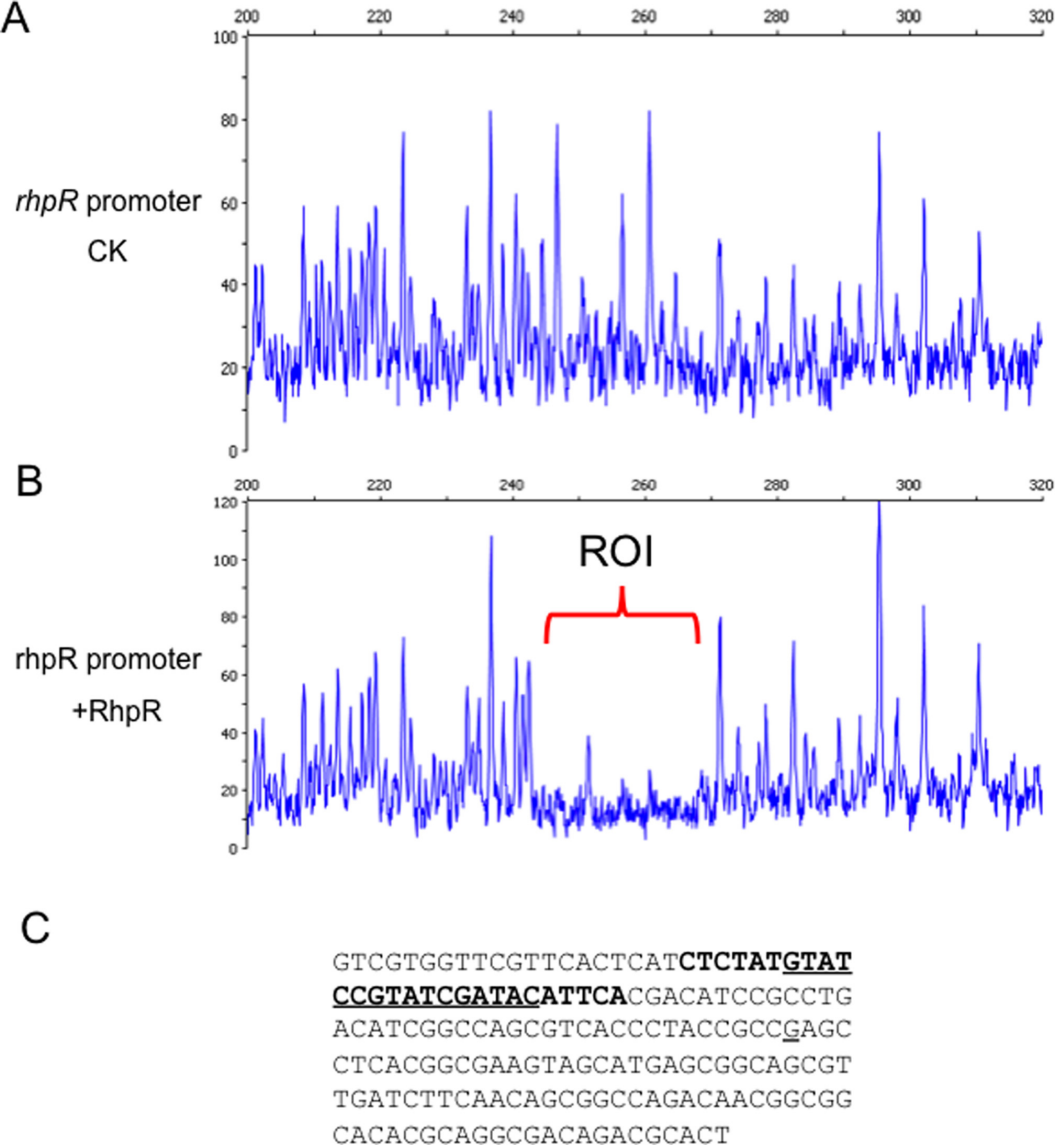
RhpR directly binds to the IR motif in its own promoter. Electropherograms showing the protection pattern of the *rhpR* promoter after digestion with DNase I following incubation in the absence (**A**) or the presence (**B**) of 1 μM RhpR-P. ROI, region of interest. (**C**) The *rhpR* promoter sequence (−173 ∼ −1 from ATG) is shown with a summary of the DNase I footprint assay results. The RhpR-protected region is in boldface, and the IR motif is in boldface and underlined. The underlined G represents the transcription start site.

To define the RhpR-binding site in its own promoter DNA, we performed a dye-based DNase I footprint assay by using dye primer sequencing on the 3730 DNA Analyzer (Applied Biosystems), which pinpointed the IR motif (GTATCGTATCGATAC, from −147 to −132) in the middle of this protected region (Figure 2) (23). The phosphorylation state of RhpR did not change the protected site sequence in the promoter DNA (data not shown).

### RhpR accepts phosphate groups from the phosphodonor acetyl phosphate

**Figure 3.**
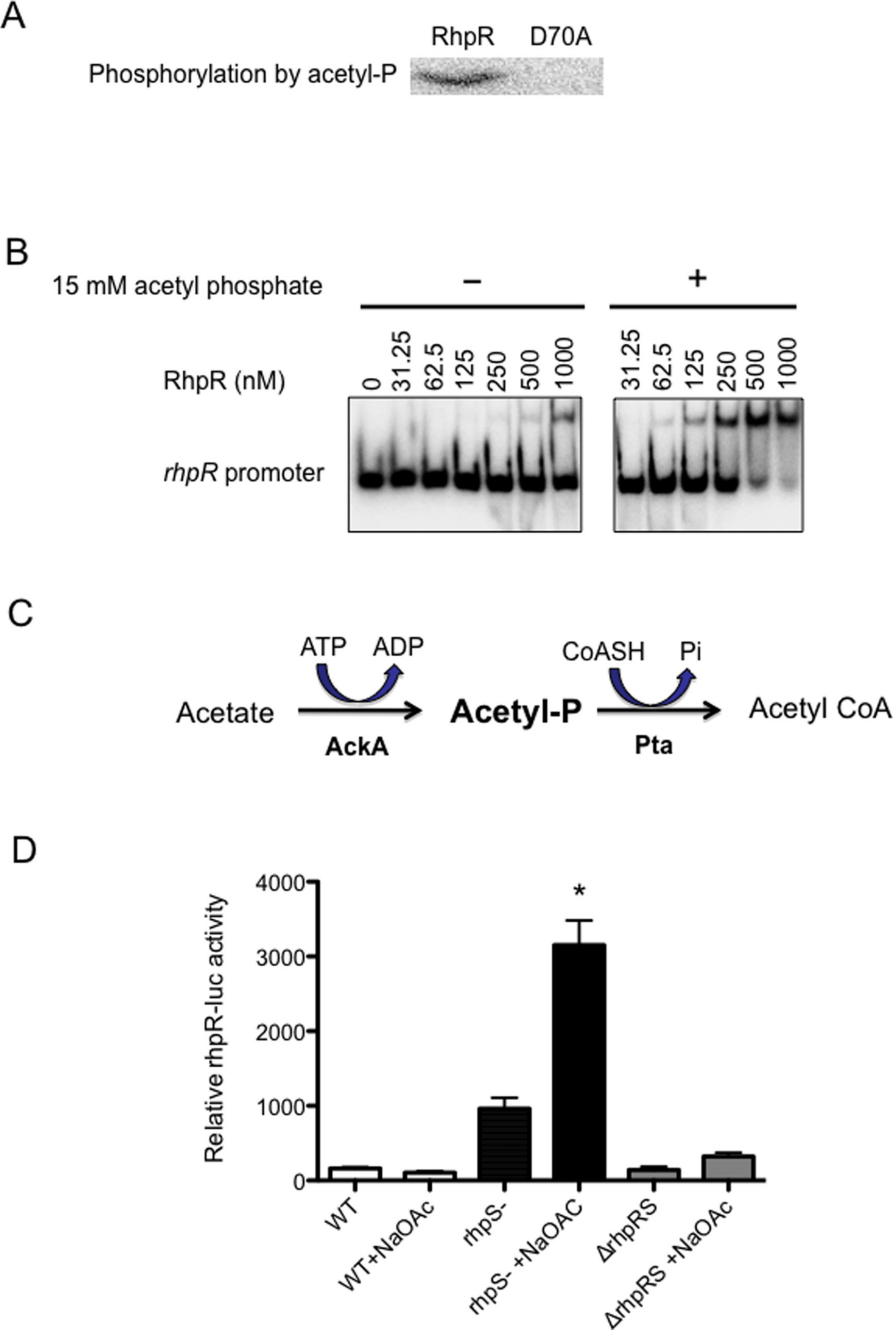
RhpR is phosphorylated by acetyl phosphate, whose substrate acetate activates *rhpR* in the absence of *rhpS*. (**A**) RhpR (10 μM), but not RhpR-D70A, was phosphorylated by [γ-^32^P]-acetyl phosphate. (**B**) The DNA binding activities of RhpR to the *rhpR* promoter in the absence (left panel) or the presence (right panel) of acetyl phosphate are shown. (**C**) The diagram shows the ACK-PTA pathway in *Pseudomonas syringae*. PSPTO_1669 (*ackA*) encodes acetate kinase (AckA), which catalyzes acetate to acetyl phosphate. PSPTO_1169 (*pta*) encodes phosphate acetyltransferase (Pta) that synthesizes acetyl-CoA from acetyl ∼ P and CoASH. (**D**) The effect of acetate supplement is shown for the relative *rhpR-luc* activities in the wild-type, the *rhpS* mutant and the Δ*rhpRS* mutant grown in MM. The asterisk denote statistical significance between rhpS- and rhpS- +NaOAc; * *P* < 0.05.

Acetyl phosphate is the primary small phosphodonor for a wide range of TCSs (30–31,39). We tested whether acetyl phosphate can efficiently phosphorylate RhpR. [γ-^32^P]-acetyl phosphate was synthesized as described in the ‘Materials and Methods’ section. Asp_70_ is the putative phosphorylation site that is required for RhpR activity (5,23). Purified recombinant RhpR and RhpR-D70A, the mutant RhpR with Asp_70_ replaced by Ala, were incubated with [γ-^32^P]-labeled acetyl phosphate in the reaction buffer at 37°C for 30 min. The phosphorylated RhpR was readily detected, while RhpR-D70A was not be phosphorylated by acetyl phosphate (Figure 3A). Like the addition of both RhpS^C^ and ATP, acetyl phosphate also induced an ∼8-fold increase in the DNA binding affinity of RhpR, as determined by EMSA (Figure 3B).

### Acetate activates RhpR in the absence of RhpS

Acetyl phosphate is an intermediate in the acetate kinase (Ack)-phosphate acetyltransferase (Pta) pathway in most bacterial species including *P. syringae* (40). After diffusing freely through the bacterial membrane, acetate can be converted to acetyl phosphate and then acetyl-CoA via the enzymes Ack and Pta, respectively (31) (Figure 3C). Thus, adding acetate to the medium can increase intracellular levels of acetyl phosphate, which in turn may activate some TCSs (39,41). In light of these data, we sought to determine whether acetate plays a role in RhpR activation *in vivo*. The wild-type strain, the *rhpS* mutant and the Δ*rhpRS* strains carrying a *rhpR-luc* reporter were cultivated in KB medium before transferred into MM supplemented with or without 10-mM sodium acetate. After 4 h, sodium acetate induced the expression of *rhpR-luc* by about 3-fold in the *rhpS* mutant, but not in the wild-type or the Δ*rhpRS* mutant (Figure 3D). These results suggest that acetyl phosphate acts as a physiological phosphodonor to RhpR.

### RhpS^C^ confers phosphatase activity toward P-RhpR

**Figure 4.**
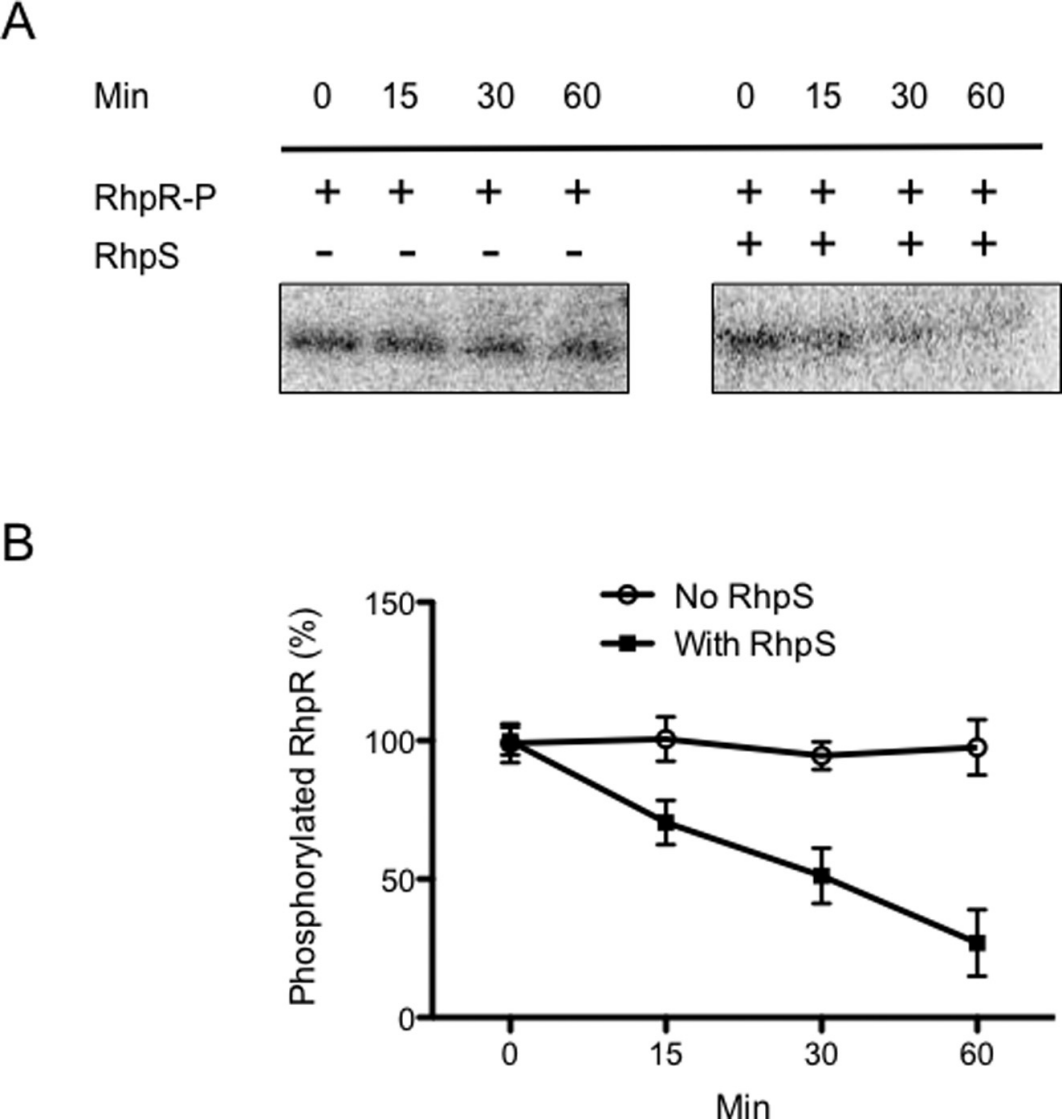
RhpS^C^ confers phosphatase activity toward P-RhpR. (**A**) RhpR (50 μM) was phosphorylated by [γ-^32^P]-acetyl phosphate. [γ-^32^P]-RhpR was mixed with or without 5 μM RhpS^C^ and incubated at room temperature for 1 h. Aliquots of 10 μl were removed from the reaction mixtures at different time intervals and quenched by the addition of 10 μl 2x SDS loading buffer. Samples were analyzed by 13% SDS-PAGE and autoradiography. (**B**) The results from (A) were quantified by Quantity One (Bio-Rad). The error bars represent the standard deviations calculated from two independent experiments.

RhpR requires phosphorylation to repress the T3SS genes, and the presence of RhpS reverses the repression (5,23). As many sensor kinases have dual enzymatic activities of kinases as well as phosphatases (42), we hypothesized that RhpS acts as a phosphatase that dephosphorylates P-RhpR. To address this, we performed *in vitro* phosphatase assays using acetyl phosphate-phosphorylated RhpR and RhpS^C^. RhpR was pre-phosphorylated by [γ-^32^P]-acetyl phosphate *in vitro* ([γ-^32^P]-RhpR), incubated with purified RhpS^C^, and loss of the phosphoryl group was monitored over a 60-min time-course. [γ-^32^P]-RhpR incubated in the absence of RhpS^C^ was used as a control to account for spontaneous dephosphorylation. Dephosphorylation of [γ-^32^P]-RhpR was observed when RhpS^C^ was present, while spontaneous [γ-^32^P]-RhpR dephosphorylation barely occurred in the absence of RhpS^C^ during a 60 min time-course (Figure 4, A and B). These data demonstrated that RhpS^C^ directly dephosphorylates P-RhpR.

### Genome-wide analysis of the RhpR-binding regions by ChIP-seq

Our previous studies identified a few RhpR-regulated genes by searching for the putative IR motives in the *P. syringae* genome (23), but these genes did not explain the biological role of RhpRS and the signaling connection to the T3SS cascade. To better understand the RhpRS function, we sought to globally characterize RhpR-binding loci on the chromosome of *P. syringae* using the ChIP-seq (35).

**Figure 5.**
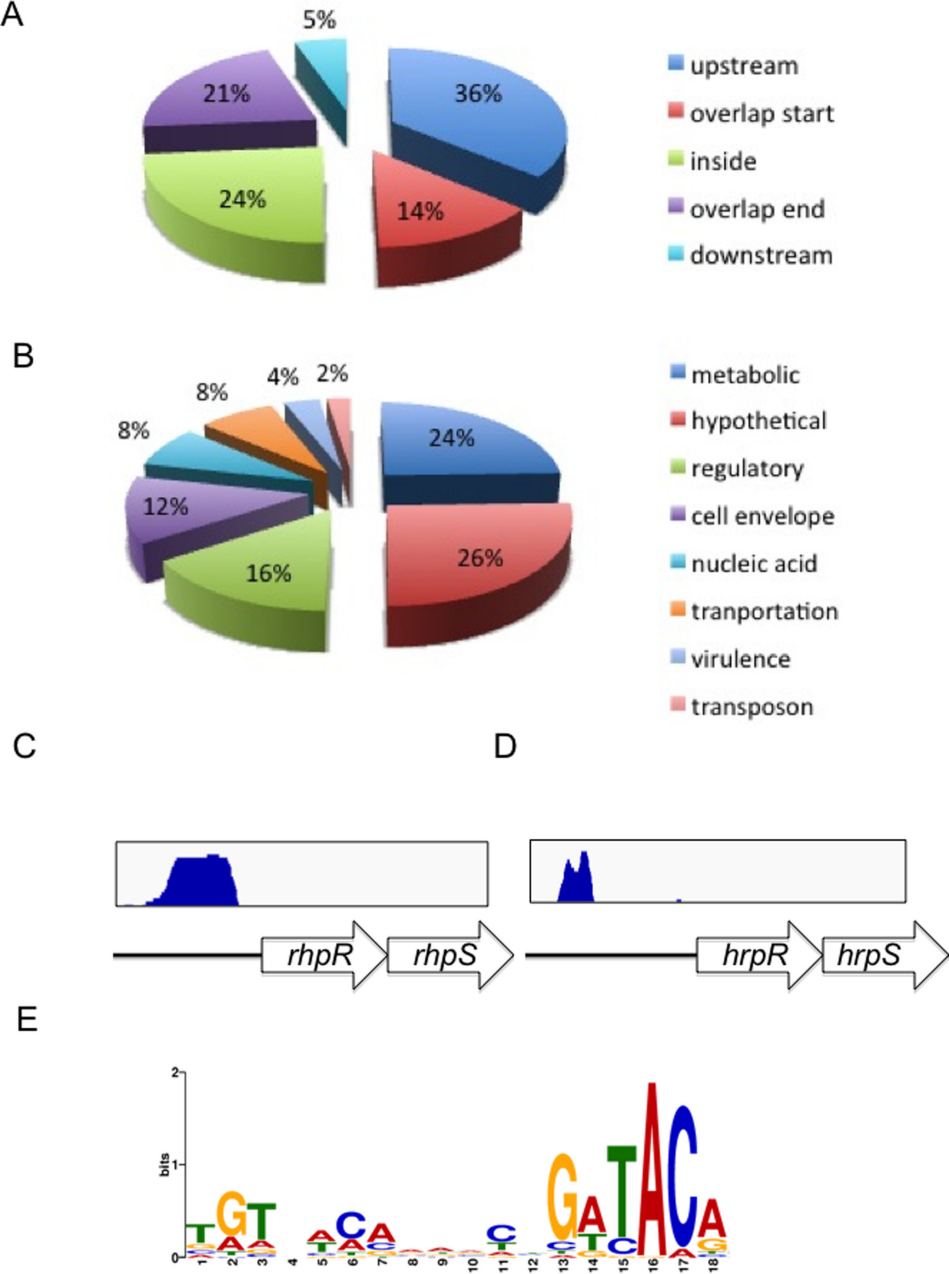
ChIP-seq reveals 167 *invivo* binding sites of RhpR. (**A**) The positions of the RhpR-binding peaks are represented in a pie chart. (**B**) The pie charts display the percentage of RhpR targets with functional categories. (**C**) and (**D**) Original sequence peaks show the RhpR binding regions in the *rhpR* and *hrpR* promoters. (**E**) The most highly significant motif identified by ChIP-seq using the MEME tool is shown (38). The height of each letter represents the relative frequency of each base at each position in the consensus sequence. Positions that are perfectly conserved contain two bits of information.

Human influenza hemagglutinin (HA)-tagged full-length RhpR was expressed under its own promoter in the pHM2 vector in the *rhpS* mutant. The HA-IP efficiency was much higher in the *P. syringae pv. phaseolicola* strain than the *P. syringae pv. tomato* strain (data not shown), so we used the former strain and HA-tagged RhpR_psph_ in our ChIP-seq analysis. Sequence reads were obtained from two independent ChIP-seq assays using an HA-specific antibody and mapped to the *P. syringae pv. phaseolicola* genome. Using MACS software (37), we identified 167 enriched loci (*P*-value = E-5) harboring RhpR-binding peaks (Supplementary Table S2) that were enriched by >2-fold, and these were absent in the control samples using the wild-type strain without the HA-tag. These 167 loci are distributed throughout the genome in intergenic region (76%) and within coding regions (24%). Notably, 50% of all peaks were located upstream of genes (36%) or overlapped with the promoter regions of genes (14%) (Figure 5A).

Genes identified by ChIP encode proteins with diverse functions, including those involved in metabolism (24%), virulence (8%), regulatory proteins (16%), cell envelope (12%), transporters (8%), nucleic acids (8%), transposons (4%) and hypothetical proteins (26%) (Figure 5B). As expected, RhpR binds its own promoter that contains the IR motif (Figure 5C). Significantly, RhpR directly binds the *hrpR* promoter (Figure 5D), suggesting the direct regulation of *hrpRS* operon by RhpR. A major binding site was found 958 bp upstream of the translational start codon of *hrpR* that contains an imperfect IR motif (ATTTCAACGCTGATAC). Interestingly, the binding site was also found upstream or inside of several T3SS effector genes including *avrE*, *hopG1* and *hopR1*. These findings suggested that RhpR regulates the T3SS genes at different levels. Binding sites were also detected upstream or inside of 11 genes encoding conserved regulatory proteins with unknown functions (Supplementary Table S2).

In addition, more than a third of the RhpR-binding sites are located on genes involved in nucleic acids (such as *gyrB*, *rpoD* and *dnaG*) and metabolic functions (such as *sdhD* and *nuoN*). Interestingly, genes encoding type 4 pilus-associated proteins and other cell envelope-related genes (such as flagella-, porin-, lipoprotein- and cell wall-related genes) are highly represented in the list of genes containing binding sites, suggesting the possible regulation of RhpR in the cell envelope. Collectively, these newly identified target genes suggested that RhpR regulates multiple cellular functions.

**Figure 6.**
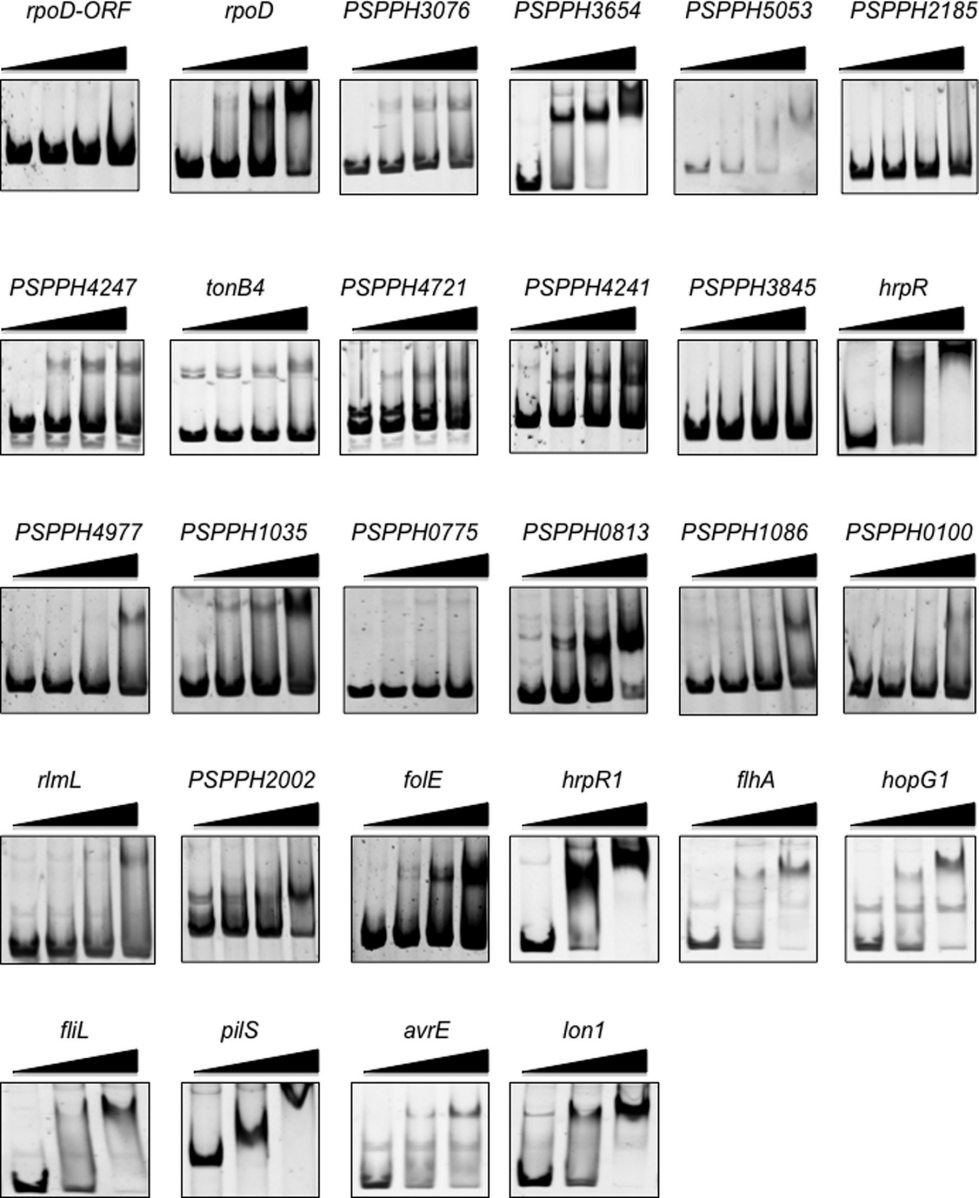
*Invitro* EMSA verification of the ChIP-seq data. Twenty-eight top RhpR-binding regions (PCR-amplified from *P. syringae pv. phaseolicola*) identified by ChIP were PCR amplified (primers are listed in Supplementary Table S1) before mixed with 0, 0.25, 0.5 or 1 μM (0, 0.5 or 1 μM for three-lane panels) RhpR_psph_ for the EMSA assay.

Using the MEME suite, an 18-bp RhpR-binding consensus sequence (TGTN[T/A][C/A]N_6_GATAC[A/G], *P*-value = 1.3e-31) was identified in 151 of 167 peaks (Figure 5E, Supplementary Table S2) (38), and this sequence matches the motif revealed in the aforementioned footprint assay (Figure 2). To validate the ChIP-seq data, we ranked the peaks by the enrichment fold. We then picked the top 20 targets and 6 additional genes (*hopR1, flhA, hopG1, fliL, pilS* and *avrE1*) involved in virulence and subjected them to EMSA and qRT-PCR. The results are summarized in Table 1. RhpR was able to specifically bind to all but four (PSPPH2185, *tonB4*, PSPPH3845 and PSPPH0775) of the ChIP-seq peaks tested (Figure 6), and directly regulates 16 of these genes. As expected, we saw a positive relationship between enrichment fold and validation in EMSA. More mismatches were observed in the first half than in the second half of conserved IR motifs, consistent with our previous finding showing that the second half is more important (23). Based on the qRT-PCR assay, *rhpR* largely regulated its downstream genes, including the T3SS genes *hrpR*, *hopR1* and *avrE1*.

**Table 1.** Top RhpR-binding genes

Fold enrichment	Gene	Function	Position	Category	IR sequence	EMSA	Distance to ATG	Fold-change in rhpS^−^
3.86565	*rpoD*	RNA polymerase, sigma 70	overlap end	nucleic acid	GTATCN6GACAC	Y		NS
3.77545	PSPPH3076	hypothetical	upstream	hypothetical	GTAACN6GACAC	Y	−100	−3.6 ± 1.1
3.74968	PSPPH3654	TetR family transcription factor	upstream	regulatory	GTATAN6GTTAC	Y	−40	NS
3.66732	PSPPH5053	aminotransferase	downstream	metabolism	GTACCN6GATAC	Y		−2.7 ± 0.4
3.60794	PSPPH2185	histidine kinase	upstream	regulatory	ATTACN6GGCAC	N	−421	NS
3.46699	PSPPH4247	GAF/GGDEF/EAL protein	upstream	regulatory	GTAACN6CATAC	Y	−145	−2.5 ± 0.5
3.43841	*tonB4*	siderophore transporter	upstream	transporter	GTTCCN6CTTAC	Y	−211	−2.7 ± 0.4
3.4234	PSPPH4712	anti-CoA dehydrogenase	overlap end	metabolism	GTAACN6GATGC	Y		−4.6 ± 0.7
3.36312	PSPPH4241	LuxR family transcription factor	upstream	regulatory	GTTTCN6GATAC	Y	−50	−6.0 ± 3.0
3.36139	PSPPH3845	arsC family protein	upstream	metabolism	GTGACN6GGTAC	N	−87	NS
3.32106	*hrpR*	T3SS	upstream	virulence	ATTTCN6GATAC	Y	−958	Abolished (5)
3.31158	PSPPH4977	GNAT family acetyltransferase	inside	metabolism	ATATCN6GATAC	Y	239	+3 ± 1.0
3.23885	PSPPH1035	lipoprotein	upstream	metabolism	GAAGCN6GATAC	Y	−121	NS
3.20849	PSPPH0775	chaperone protein	inside	transporter	AGATCN5GTTAC	N	340	NS
3.20031	PSPPH0813	hypothetical	upstream	hypothetical	GTTTCN6GATAC	Y	−120	−3.3 ± 1.0
3.19561	PSPPH1086	lipoprotein	upstream	hypothetical	GCAAAN6GATAC	Y	−90	NS
3.18272	PSPPH0100	recombinase	inside	nucleic acid	GGATTN6GATAC	Y	732	NS
3.12415	*rlm*L	23S rRNA methyltransferase	overlap end	metabolism	TGTACN6GTTAC	Y		NS
3.11668	PSPPH2002	Ca binding protein	upstream	regulatory	GTATCN6CGTAC	N	−72	NS
3.11231	*folE*	GTP cyclohydrolase	upstream	metabolism	GTAAGN6GATAC	Y	−41	−2.1 ± 0.1
2.88548	*hopR1*	T3SS effector	inside	virulence	GTATGN6GATAC	Y	242	−2.2 ± 0.6
2.63489	*flhA*	flagellar biosynthesis protein	upstream	cell envelop	GCAACN6GATAC	Y	−59	−2.6 ± 0.9
2.35805	*hopG1*	T3SS effector	overlap end	virulence	CCTACN6GTTAC	Y		−17.1 ± 1.6
2.33503	*fliL*	flagellar basal body protein	upstream	cell envelop	GATTCN6GACAC	Y	−76	NS
2.28869	*pilS*	two-component system	inside	regulatory	ATGTCN6GGTAC	Y	437	−3.6 ± 1.4
2.20673	*rhpR*	TCS	upstream	virulence	GTATCN6GATAC	Y	−69	+>10 (23)
2.17765	*avrE1*	T3SS effector	inside	virulence	GACATN6GTTAC	Y	3127	−7.6 ± 3.0

Mismatches are underlined in IR sequences. Y: positive in EMSA; N: negative in EMSA; NS: no significance in qRT-PCR.

### Microarray analysis of RhpR-regulon in KB and MM

Molecular genetic studies indicate that upon mutation of *rhpS*, *rhpR* is strongly induced in KB and MM possibly via an autoactivation mechanism (5,23). Therefore, to identify the RhpR regulon and to determine the effect of environmental conditions on the profile of RhpR regulon, microarray analyses were conducted to compare the gene expression profiles in *rhpS* mutant and *ΔrhpRS* mutant in KB and MM. ORFs that showed a >1.5-fold (log_2_ [1.5] ≈ 0.585) difference were considered to be differentially expressed. Overall, 102 ORFs upregulated (Supplementary Table S3) and 145 ORFs downregulated (Supplementary Table S4) by *rhpR* when the bacteria were grown in KB. In MM, however, 519 ORFs were upregulated (Supplementary Table S5) and 567 ORFs were downregulated (Supplementary Table S6) by *rhpR*.

Of the 102 genes upregulated by RhpR in KB, three encode proteins with transcriptional regulatory functions. Forty-seven genes encode ribosome-related proteins or are in linkage disequilibrium with ribosomal genes, suggesting a key role of RhpR in promoting protein synthesis in the nutrient-rich condition. Six genes were flagella-related, implying a role of RhpR to stimulate flagella formation or cell movement. Ten other genes encoded proteins with envelop-related functions, including five transporter genes, two lipoprotein genes, one lipopolysaccharide biosynthesis gene and one type IV pilus biogenesis gene. The remaining genes upregulated by RhpR in KB belong to various other functional categories.

In KB, 145 genes were downregulated by RhpR. Interestingly, 24 of these genes were located on the pDC3000A and pDC3000B plasmids, and at least 11 of the plasmid genes were involved in plasmid mobilization and partition. In addition, 15 genes dispersed in the chromosome encode transposases or site-specific recombinases. These results implied a role of RhpR in regulating the stability of the bacterial genome. PSPTO_1205 and PSPTO_2310 encoded a putative ribosomal subunit interface protein and a ribosome modulation factor-related protein, respectively. Reduced expression of these two regulatory genes may contribute to the increased expression of other ribosomal genes in KB. A cluster of 7 RhpR downregulated genes that encode siderophore yersiniabactin synthesis and transport were also found. Yersiniabactin plays a role in self-protection against heavy metals such as copper (43). It can also enhance bacterial pathogenicity by chelating iron, leading to iron deficiency in the host cells (44). Nine genes encoded chaperonin-like proteins, including four heat shock proteins, two chaperonins, one cold shock protein, DnaJ and DnaK. These genes are often induced under stress conditions and the corresponding proteins have a protective role against environmental stresses (45). Fourteen genes had cell wall-related functions, including two involved in alginate synthesis, two levansucrases, two lipoproteins, six transporters and two putative membrane proteins. Six of the downregulated genes encoded T3SS-related proteins. Forty-four genes were involved in metabolism. Six genes encoded regulatory functions. The remaining genes downregulated by RhpR in KB were involved in various other functions.

Of the 519 ORFs upregulated by RhpR in MM, 49 had regulatory functions, including *rhpR* itself. Consistent with the large number of regulatory genes, the RhpR-induced genes in MM displayed a wide range of diverse functions. A cluster of 16 genes were related to flagella and chemotaxis. These genes are also induced by different levels by RhpR in KB, suggesting a role of RhpR in stimulating cell movement in both growth conditions. Interestingly, 12 genes induced by RhpR in MM were inhibited by RhpR in KB. These include a cluster of three genes related to yersiniabactin synthesis. Notably, only four ribosomal genes were induced by RhpR in MM, which is in striking contrast with the large number of ribosomal genes induced by RhpR in KB.

Of the 567 ORFs downregulated by RhpR in MM, 55 encoded the T3SS-related proteins, which is consistent with our previous observation that RhpR is a suppressor of the T3SS (5). *hrpR*, *hrpS* and many other known T3SS-related genes were also downregulated by RhpR, but did not meet by the 1.5-fold cutoff criteria. Seventy-two genes encoded transporters for various substrates, implicating RhpR in reducing membrane permeability when nutrients are limited. Thirty of the RhpR-inhibited genes in MM were ribosomal genes or closely linked to ribosomal genes. Most of the ribosomal genes were induced by RhpR in KB. These results suggested that RhpR alters its role to modulate protein synthesis in response to nutrient conditions, i.e. RhpR protein synthesis is inhibited when nutrients are poor but increased under nutrient-rich conditions. Thirty-eight genes were located on the pDC3000A and pDC3000B plasmid, and 11 of these genes were related to plasmid mobility and partitioning. Many of these genes were also suppressed by RhpR in KB, suggesting that RhpR regulates plasmid stability. Fifty-two transposases and transposition-related genes were inhibited by RhpR in MM. These genes were different from the transposition-related genes inhibited by RhpR in KB. Nonetheless, downregulation of these genes by RhpR suggests that RhpR plays a role in stabilizing the bacterial genome. Five genes encoded chaperonin-related proteins, and each of the five genes were also inhibited by RhpR in KB.

## DISCUSSION

While the T3SS of *P. syringae* is induced during interaction with the plant or culture in MM (11), how the bacterium senses these environments to activate the T3SS remains largely unknown. Previously, we have identified a TCS RhpRS that regulates *P. syringae* T3SS genes (5,23). *rhpS* is required for full induction of *P. syringae* T3SS genes *in plants* as well as in MM and pathogenicity on host plants. Interestingly, deletion of the *rhpRS* loci largely recovers the induction of T3SS genes to a level similar to that in the wild-type strain and restores pathogenicity on host plants. This is echoed by the overexpression of RhpR in the *ΔrhpRS* deletion strain that dramatically inhibits the induction of T3SS genes, suggesting RhpR as a repressor of T3SS genes (5). Subsequently, we have demonstrated that RhpR autoactivates its own promoter (23). Promoter deletion and mutagenesis analyses revealed the IR element, GTATC-N_6_-GATAC, which is crucial for the RhpR-dependent induction. The following genome-wide bioinformatic search for the putative IR elements revealed that seven genes are regulated in an RhpR-dependent manner (23).

Although our previous genetic results strongly suggested that RhpS tunes the phosphorylation state of RhpR en route to regulate T3SS genes, biochemical evidences were missing to directly support the hypothesis. In this study, we provide biochemical results that illustrate the molecular mechanism of RhpRS in mediating the T3SS gene regulation. As shown by a phosphorylation assay, RhpS^C^ confers strong autokinase activity, and efficiently phosphorylates RhpR. The EMSA and footprint assay proved that RhpR binds to the IR motif in its own promoter, which was proposed by our previous paper (23). More importantly, the EMSA assay directly demonstrated that the phosphorylated RhpR has much higher affinity to target DNA than the unphosphorylated RhpR. This result is consistent with our previous observation showing that RhpR-D70A mutant is incapable of repressing the T3SS genes (5).

It's been reported that response regulators can be phosphorylated by unrelated sensor kinases or by small phosphate donor molecules such as acetyl phosphate or carbamoyl phosphate in the absence of their cognate sensor kinases (31). As expected, in the absence of RhpS, RhpR is phosphorylated by other phosphate donors, thus inhibits the induction of T3SS genes *in planta* or MM. In the present paper, an EMSA experiment showed the addition of acetyl phosphate indeed increased the DNA-binding affinity of RhpR, suggesting acetyl phosphate-mediated phosphorylation of RhpR. The DC3000 genome carries a putative acetate kinase (PSPTO_1669) involved in acetyl-phosphate production. In order to confirm its enzymatic function, we purified the recombinant PSPTO_1669 protein and found it able to catalyze the formation of [γ-^32^P]-labeled acetyl phosphate. Moreover, a supplement of acetate to the MM significantly induced the *rhpR* transcription in the *rhpS* mutant. Nevertheless, we could not rule out the possibility that other noncognate sensor kinases are involved in RhpR phosphorylation.

In the T3SS-inducing conditions, RhpS is thought to function as a phosphatase to keep RhpR in the unphosphorylated state that is not capable of inhibiting the induction of T3SS genes (5). This speculation can be supported by the lack of acetate-mediated induction of *rhpR-luc* in the wild-type strain. Furthermore, a phosphatase assay demonstrated that RhpS^C^ efficiently dephosphorylates P-RhpR. Although the nature of signal that RhpS senses to turn on its phosphatase activity in the T3SS-inducing condition still remains elusive, the phosphatase function of sensor kinases has been reported in many TCSs including QseBC, CovSR and VanSR (46–48).

Previously we searched for IR-containing promoters in the DC3000 genome, which identified 20 candidates with one mismatch. However, the subsequent northern analysis revealed that only five of those genes were real RhpR targets (23). In order to identify all the *in vivo* binding sites of RhpR, we have developed a ChIP-seq method, and revealed 167 binding sites. Most of the peaks contain an IR-like motif (GTATCN_6_GATAC). The majority of the genes (42%) are involved in metabolism and nucleic acid, indicating the profound roles of RhpRS in regulating a variety of metabolic pathways. Other major RhpR targets included a group of transcription factors and virulence factors. EMSA confirmed that RhpR binds to 24 of 28 selected sites, demonstrating the high accuracy of the ChIP-seq data. The most important finding of the ChIP-seq is the direct interaction between RhpR and the *hrpR* promoter, which contains a putative IR-motif (ATTTCAACGCTGATAC) at 958 bp upstream of the start codon (109 bp downstream of the upstream gene PSPPH1269). Unlike a typical repressor that binds to a promoter region close to the translation start site, the binding of RhpR on the *hrpR* promoter may interfere with other regulators of *hrpR*, such as HrpA and GacA. Previously we could not detect the binding of RhpR to an *hrpR* promoter region using a ChIP-PCR approach (23). We reasoned that the previously tested promoter region is ∼800 bp downstream of the IR motif, and is beyond the RhpR-binding region. Our ChIP-seq results provide a useful database for further characterization of RhpR-regulated functions in the future.

Beside T3SS, whether other cellular functions are also tuned by RhpRS remains largely unknown. The genome-wide identification of the *rhpR* regulon is critical for our understanding of the biological processes associated with this TCS. To this end, we conducted whole genome microarray analyses of DC3000 to determine genes regulated by *rhpR* in either KB or MM, and found that *rhpR* regulates more than 1000 of non-T3SS genes involved in multiple functional categories, including cell envelope, transporters, transposases, metabolism, gene regulation and ribosome proteins. The largest categories in RhpR regulon are ribosomal genes and genes involved in cell envelope, suggesting that RhpR regulates protein synthesis and functions of cell envelope. The identification of a group of transposases in the RhpR regulon is echoed by our previous microarray assay showing that *hrpRS* negatively regulates 54 transposase genes in MM, indicating a possible link between mobile elements and pathogenicity (32). The microarray analyses strongly indicate that *rhpR* is a global regulator that exerts a profound influence on many aspects of biological processes in *P. syringae*.

**Figure 7.**
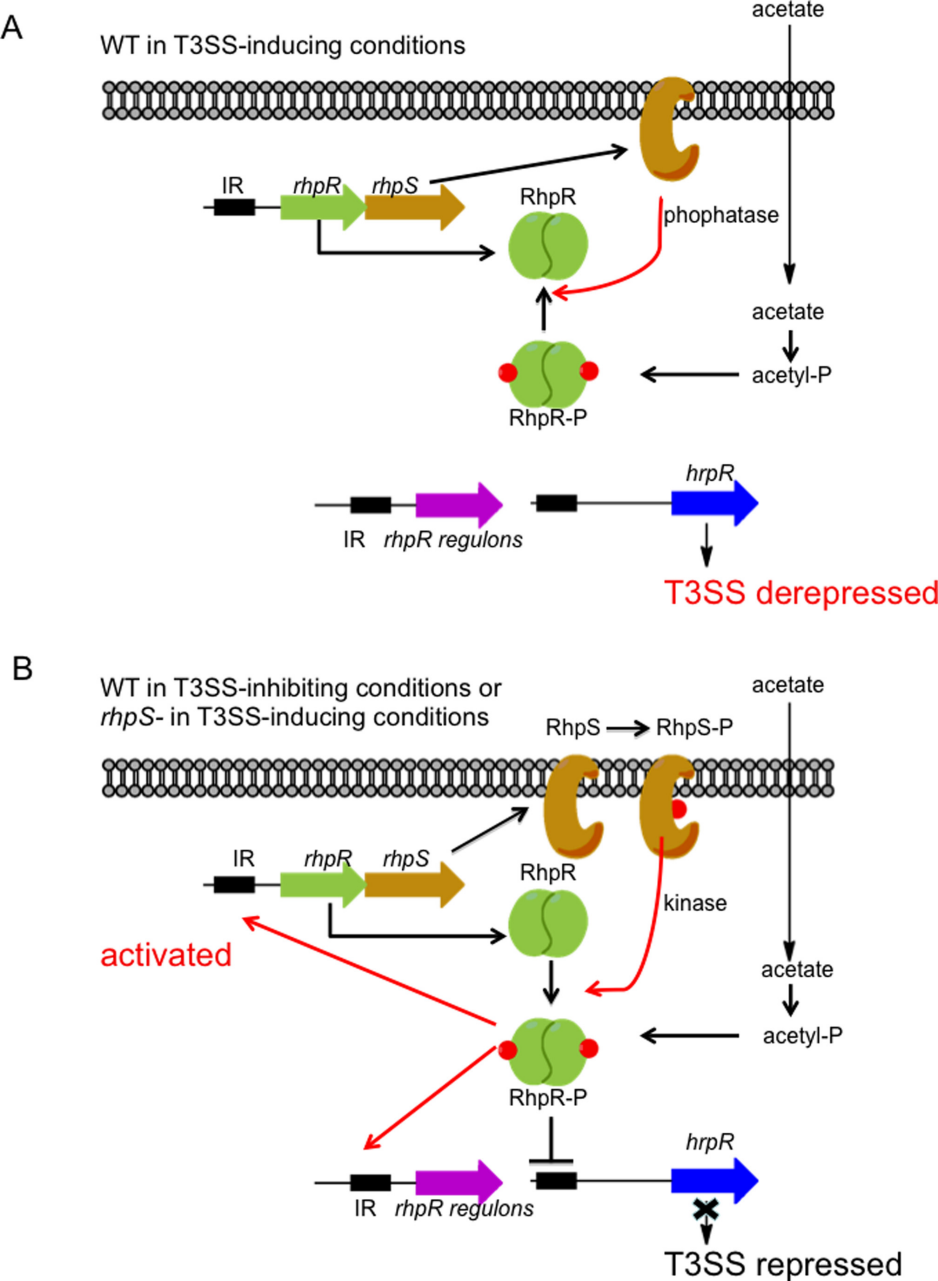
Proposed models of RhpRS-mediated regulation of T3SS genes in *Pseudomonas syringae.* (**A**) When the wild-type strain is grown in T3SS-inducing conditions (*in planta* or MM), RhpS preferably functions as a phosphatase that keeps RhpR in an unphosphorylated state. RhpR is expressed at a low level, which allows the *hrpRS-hrpL*-T3SS cascade to be activated. (**B**) In the absence of RhpS as a phosphatase, RhpR is phosphorylated by RhpS as a kinase or acetyl phosphate and remains in the phosphorylated state. Compared to RhpR, RhpR-P binds more tightly to its own promoter carrying an IR motif, which in turn activates its own promoter and produces more P-RhpR. Subsequently, a high level of P-RhpR directly represses *hrpRS*, thus compromising T3SS genes. A supplement of acetate in the MM can further induce this process in the absence of RhpS.

Based on all the genetic, biochemical, genomic and transcriptomic analyses on *rhpRS* in the present and previous papers, here we propose a model of its complicated regulatory mechanism on the T3SS genes (Figure 7). When the wild-type strain is grown in T3SS-inducing conditions, RhpS mainly functions as a phosphatase that keeps RhpR in an unphosphorylated state. The unphosphorylated RhpR is incapable of activating its own promoter, and thus the *rhpR* gene is expressed at a low level in the wild-type strain. The low level of RhpR is unable to repress the *hrpR* promoter via binding to the IR motif in the promoter, and thereby, the *hrpRS* operon is derepressed and subsequently activates *hrpL* and other T3SS genes (Figure 7A). In the absence of RhpS protein, RhpR is phosphorylated by small molecule phosphate donors such as acetyl phosphate or by yet to be identified noncognate sensor kinases. P-RhpR binds more tightly to the IR motif in its own promoter, which in turn autoactivates its own expression. Subsequently, the highly phosphorylated RhpR binds the *hrpR* promoter and represses the T3SS cascade (Figure 7B).

RhpR directly binds to 167 loci on the chromosome, and tunes almost a fifth of the genome. Our study demonstrates that RhpRS is not only a master regulator of T3SS, but also a global regulator of multiple pathways in *P. syringae.*

## ACCESSION NUMBERS

Microarray and ChIP-seq data are available in the National Center for Biotechnology Information Gene Expression Omnibus under series GSE15032 and GSE58533, respectively.

## SUPPLEMENTARY DATA

Supplementary Data are available at NAR Online.

SUPPLEMENTARY DATA
