# Supplementary material for: Molecular mechanisms of two-component system RhpRS regulating type III secretion system in Pseudomonas syringae
Source: Nucleic Acids Res. 2014 Sep 23;42(18):11472–86. doi: 10.1093/nar/gku865 (PMC4191427; doi:10.1093/nar/gku865)
Supplement: SUPPLEMENTARY DATA [file supp_42_18_11472__index.html]

Molecular mechanisms of two-component system RhpRS regulating type III secretion system in Pseudomonas syringae — Molecular mechanisms of two-component system RhpRS regulating type III secretion system in Pseudomonas syringae — SUPPLEMENTARY DATA 

# Molecular mechanisms of two-component system RhpRS regulating type III secretion system in *Pseudomonas syringae*

## SUPPLEMENTARY DATA

**Files in this Data Supplement:**

- SUPPLEMENTARY DATA
- SUPPLEMENTARY DATA
