# Supplementary material for: Molecular mechanisms of two-component system RhpRS regulating type III secretion system in Pseudomonas syringae
Source: Nucleic Acids Res. 2014 Sep 23;42(18):11472–86. doi: 10.1093/nar/gku865 (PMC4191427; doi:10.1093/nar/gku865)
Supplement: SUPPLEMENTARY DATA [file supp_gku865_nar-01035-z-2014-File010.docx]

**Molecular mechanisms of two-component system RhpRS regulating type III secretion system in *Pseudomonas syringae***

Xin Deng^1#^, Haihua Liang^2#^, Kai Chen^1^, Chuan He^1^, Lefu Lan^3*^, Xiaoyan Tang^4*^

**Figure S1.** Regulatory network of T3SS in *P. syringae*. RhpR/RhpS and AefR positively regulate the transcription of *hrpRS*. GacS/GacA activates the transcription of *hrpRS* and *rpoN*. HrpS is repressed by HrpV via protein–protein interaction. HrpG interacts with HrpV and derepresses HrpS. HrpS and HrpR proteins form heterodimer. HrpR protein is degraded by Lon protease. HrpR and HrpS associate with RpoN in the *hrpL* promoter to activate *hrpL* expression. HrpL recognizes the *hrp* box promoter and activates the transcription of hrp regulons. HrpA acts upstream of *hrpRS* transcription to activate T3SS gene expression.
